# Interferon Lambda: Modulating Immunity in Infectious Diseases

**DOI:** 10.3389/fimmu.2017.00119

**Published:** 2017-02-28

**Authors:** Mohammedyaseen Syedbasha, Adrian Egli

**Affiliations:** ^1^Applied Microbiology Research, Department of Biomedicine, University of Basel, Basel, Switzerland; ^2^Clinical Microbiology, University Hospital Basel, Basel, Switzerland

**Keywords:** interferon lambda, immunity, immune cells, virus, infectious diseases, bacteria, fungi, parasites

## Abstract

Interferon lambdas (IFN-λs; IFNL1-4) modulate immunity in the context of infections and autoimmune diseases, through a network of induced genes. IFN-λs act by binding to the heterodimeric IFN-λ receptor (IFNLR), activating a STAT phosphorylation-dependent signaling cascade. Thereby hundreds of IFN-stimulated genes are induced, which modulate various immune functions *via* complex forward and feedback loops. When compared to the well-characterized IFN-α signaling cascade, three important differences have been discovered. First, the IFNLR is not ubiquitously expressed: in particular, immune cells show significant variation in the expression levels of and susceptibilities to IFN-λs. Second, the binding affinities of individual IFN-λs to the IFNLR varies greatly and are generally lower compared to the binding affinities of IFN-α to its receptor. Finally, genetic variation in the form of a series of single-nucleotide polymorphisms (SNPs) linked to genes involved in the IFN-λ signaling cascade has been described and associated with the clinical course and treatment outcomes of hepatitis B and C virus infection. The clinical impact of IFN-λ signaling and the SNP variations may, however, reach far beyond viral hepatitis. Recent publications show important roles for IFN-λs in a broad range of viral infections such as human T-cell leukemia type-1 virus, rotaviruses, and influenza virus. IFN-λ also potentially modulates the course of bacterial colonization and infections as shown for *Staphylococcus aureus* and *Mycobacterium tuberculosis*. Although the immunological processes involved in controlling viral and bacterial infections are distinct, IFN-λs may interfere at various levels: as an innate immune cytokine with direct antiviral effects; or as a modulator of IFN-α-induced signaling *via* the suppressor of cytokine signaling 1 and the ubiquitin-specific peptidase 18 inhibitory feedback loops. In addition, the modulation of adaptive immune functions *via* macrophage and dendritic cell polarization, and subsequent priming, activation, and proliferation of pathogen-specific T- and B-cells may also be important elements associated with infectious disease outcomes. This review summarizes the emerging details of the IFN-λ immunobiology in the context of the host immune response and viral and bacterial infections.

## IFN-λ Expression and Signaling Pathways

**Figure 1:**
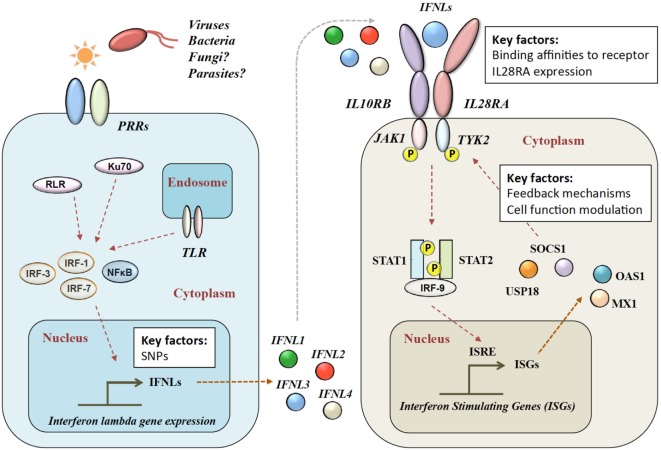
**Type III IFN signaling pathway**. Viral infection is sensed by pattern recognition receptors (PRRs), which induce IFN-λ production *via* various signaling pathways. IFN-λs bind to the heterodimeric IFN-λ receptor (IFNLR), which consists of IL28RA and IL10RB subunits. Upon binding, a JAK–STAT signaling cascade induces hundreds of IFN-stimulated genes (ISGs). RLR, RIG-1-like receptor; TLR, toll-like receptors; NF-κB, nuclear factor kappa-light-chain-enhancer of activated B cells; IL28RA, interleukin 28 receptor alpha; IL10RB, interleukin 10 receptor beta; JAK1, Janus Kinase 1; TYK2, tyrosine kinase 2; STAT, signal transducer and activator of transcription; IRF, interferon regulatory factor; ISRE, interferon-stimulated response element; MX1, interferon-induced GTP-binding protein Mx1; OAS1, 2′-5′-oligoadenylate synthetase.

Patients with infectious diseases often show heterogeneous clinical courses with a range of associated morbidities and variable mortality. This is dependent on a series of factors covering the complex aspects of host–pathogen interactions (1–5). IFNs play a crucial role in these interactions—defining the outcome of many viral, bacterial, fungal, and parasitic infections (6–16) (see Figure 1). In addition, IFNs reduce tumor cell proliferation (17, 18) and show important immune regulatory functions in autoimmunity (19, 20). These broad effects are explained through the induction of hundreds of IFN-stimulated genes (ISGs) (21). Three types of IFNs have been described, which can induce ISG expression, and add further complexity: type I with mainly IFN-αs and -βs (22–26), type II with only IFN-γ (27), and type III with IFN-λs (28–31). Although most cells can induce and release various types of IFNs, specialized immune cells are the main producers during an inflammatory process. The effects induced by single or combined IFNs in exposed cells are very heterogeneous and range from differential patterns of ISG expression, regulation of cell proliferation (18), changes in cell surface molecules such as HLA DR (32), to the maturation of monocytes to dendritic cells (33). The effects depend on the plasticity of the various IFNs involved, including the peak concentrations, concentration changes over time, binding affinities of IFNs to the specific receptors, receptor expression, potentially induced feedback mechanisms, and the target cell type itself (34).

Four IFN-λ ligands have been described: IFNL1–4, with each family member having antiviral effects on various viruses within different cell types (28). IFNL1–3 share high amino acid sequence homologies, whereas IFNL4 is more divergent with only 40.8% amino acid similarity to IFNL3 (35). The expression of IFN-λs is induced in a broad range of cell types by pattern recognition receptors including toll-like mediated (36–41), Ku70 (21398614) and RIG-1-like (24952503). Type 2 myeloid dendritic cells have been described as the main producers of IFN-λ (42–48). In mice, commonly used as a model organism for infectious disease and immune function, only IFNL2 and IFNL3 are functional, as IFNL1 and IFNL4 are present as inactive pseudogenes (49).

After release, IFN-λ binds to its heterodimeric IFN-λ receptor (IFNLR). The IFNLR consists of two subunits: α-subunit (IL28RA) and β-subunit (IL10RB) (35, 50–53). Despite high sequence homologies, binding affinities of the different IFN-λs to the IFNLR1 differ greatly. IFNL1 shows the highest binding affinity to IL28RA, and IFNL3 the lowest (54). The dimerization of the receptor subunits leads to activation of Janus Kinase 1 and tyrosine kinase 2 and phosphorylation of STAT-1 and -2, which induces the subsequent downstream signaling with the induction of hundreds of ISGs (31) (see Figure 1). IFN-α and IFN-λ both show a complex mechanism of positive and negative feedback loops, mainly modulated *via* the suppressor of cytokine signaling 1 and the ubiquitin-specific peptidase 18 (31, 55).

## IFN-λ Responsiveness to Counteract Pathogens

Two aspects are crucial to understanding the role of IFN-λs in the context of infectious diseases: (i) IFNLR distribution in infected cells and tissues and (ii) single-nucleotide polymorphisms (SNPs) in and around the genes encoding IFN-λs and IFNLR. Both aspects show important differences between humans and mice, which complicate studies and conclusions drawn from infectious disease models (56).

### IFNLR Receptor Expression

The IL10RB subunit is expressed in many cell types (57), whereas the IL28RA subunit expression is much more restricted. Expression of IL28RA mRNA has been detected in the lung, intestine, liver tissues, immune cells such as B cells, neutrophils, macrophages, and plasmacytoid dendritic cells (28, 29, 43, 58–62). Human NK cells seem not to express IFNLR (63), whereas mouse NK cells show deficient function in IL28R knockout animals (25901316). The effects of IFN-λ on cells and tissues are often measured *in vitro via* indirect markers, such as downstream expression of ISGs or changes in specific cellular phenotypes. Data on the induction of STAT phosphorylation, as the most direct measurement of signal induction, are still missing for some cell types and tissues. The IFNLR expression is regulated *via* transcription factors (31) and may show variability during an inflammatory process, which adds an additional level of complexity. Primary hepatocytes show relatively low baseline responsiveness to IFN-λs, yet upon IFN-α treatment a marked increase in IL28RA mRNA levels is observed (64, 65). Similarly, during cytomegalovirus (CMV) infection of fibroblasts, IL28RA mRNA levels increase by about twofold, but protein expression levels remain stable (66). A recent paper by Lazear et al. suggested that endothelial cells in the blood–brain barrier may be sensitive to IFN-λs, reducing permeability to West Nile virus in a mouse model (67).

Understanding which immune cells and subsets are responsive to IFN-λs in humans can be experimentally and technically challenging due to low target cell densities and less accessible cell types such as tissue resident cell types. In contrast, peripheral blood mononuclear cells (PBMCs) are relatively easy to access in order to explore responses to IFN-λs; therefore, most literature focuses on hepatocytes (from liver biopsies) and immune cells from the blood. The direct impact of IFN-λs on T-cells *via* surface expression of the specific IFNLR is subject to ongoing debate (58, 68–71). IFN-λs may also induce FOXP3-expressing regulatory T-cells (72), which may impact a series of immunoregulatory aspects during an infection as part of the inflammatory response. Several research groups confirm that IFN-λs influence the T-helper cell balance, which is shifted toward Th1 (70, 71, 73–76). The Th1/Th2 balance might be important for controlling specific infections such as helminths (6, 77, 78). In addition, the B-cell-driven humoral immune responses are also modulated by the presence of Th2 cytokines, e.g., during vaccination. We have recently shown that IFNL3 is a key regulator of the influenza virus-specific B-cell proliferation and antibody production (76). The exact mechanism of how Th1/Th2 balancing and B-cell activation is modulated by IFN-λs and how this impacts infectious disease outcome has to be explored in more detail in the future.

### Impact of SNPs

**Figure 2:**
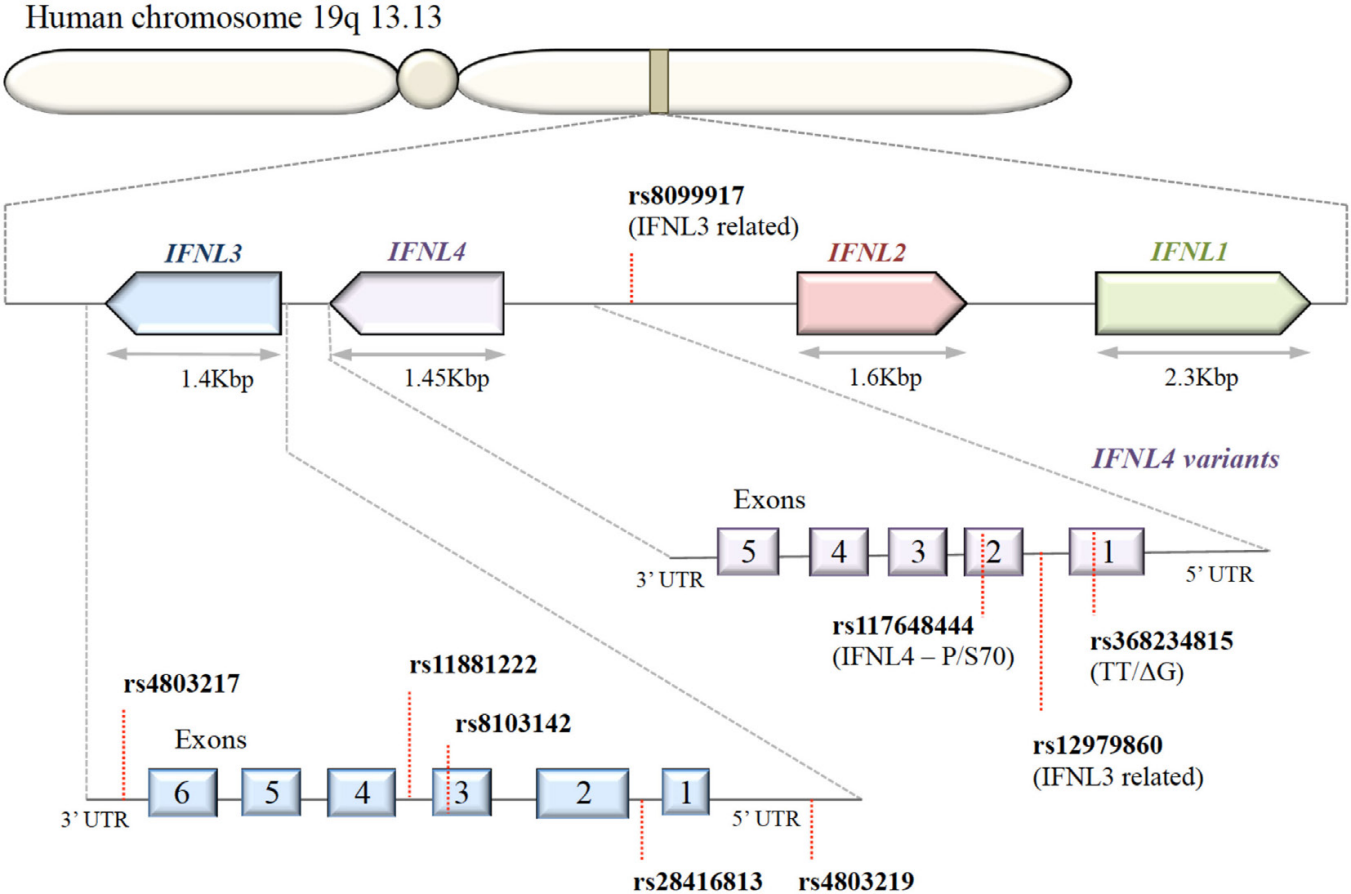
**Organization of IFNL genes in the human genome**. The IFN-λ genes are located in tandem on chromosome 19. Key single-nucleotide polymorphisms (SNPs) in coding and non-coding regions of IFN-λ genes are shown. IFNL1, IFNL2, and IFNL3 genes are functional; only a subset of the human population possess the SNP rs368234815 with ΔG frameshift mutation in exon 1, producing an in-frame IFNL4.

A series of SNPs in IFN-λ ligand and receptor genes have been described (see Figure 2). Most importantly, these SNPs have been associated with a series of important clinical phenotypes in the context of infectious diseases (see Table 1 for more details).

**Table 1:** **Single-nucleotide polymorphisms (SNPs) within the IFNL3/IFNL4 gene locus and impact on infectious diseases**.

Gene	SNP	Allele type	Effects of the allele on infectious diseases	Reference
IFNL3	rs12979860	C/T and T/T (C-major, T-minor)	HCV: decrease of effective treatment for HCV	(79, 80)
		C/T and T/T (C-major, T-minor)	HTLV1: higher proviral load and higher risk of developing HTLV-1-associated myelopathy and tropical spastic paraparesis (TSP)	(81)
		C/C (C-major)	HBV: higher inflammation and liver fibrosis in chronic hepatitis B patients	(82)
		T/T (T-minor)	EBV: observed higher level of EBV DNA in the plasma of EBV viremia patients	(83)
		T/T (T-minor)	CMV: less CMV replication in solid-organ transplant recipients	(66)
		T/T (T-minor)	CMV: lower incidence of active CMV infection and reduced CMV DNAemia in allogeneic stem cell transplant patients	(84)
		C/T and T/T (C-major, T-minor)	HSV: increased rate of HSV-1-related herpes labialis and more clinical severity	(85)
		T/T (T-minor)	ANDV: associated with mild disease progression	(86)
	rs8099917	T/G (T-major, G-minor)	HCV: lower response to PEG-IFN-α/RBV treatment	(87)
			HTLV1: high risk for developing HTLV-1-associated myelopathy and TSP	(88)
			CMV: trend to show less CMV replication in solid-organ transplant recipients	(66)
		G/G (G-minor)	ANDV: associated with mild disease progression	(86)
		T/G and G/G (T-major, G-minor)	Influenza vaccination: increased Th2 cytokine production and higher rate of seroconversion following influenza vaccination	(76)
	rs4803217	C/T (C-major, T-minor)	HCV: decreased response to PEG-IFN-α/RBV treatment	(80)
	rs10853727	A/G and G/G (A-major, G-minor)	Measles vaccination: increased post-vaccine titers against measles vaccination	(89)
	rs12980275	A/G (A-major, G-minor)	HCV: failure to clear infection (null virological response: NVR)	(80, 87)
IFNL4	ss469415590	ΔG/TT and ΔG/ΔG (frameshift variant from TT genotype)	HCV: creates a new IFNL4 gene and poorer response to PEG-IFN-α/RBV treatment	(90)
	(rs368234815)		CMV: increases susceptibility to CMV retinitis among HIV-infected patients	(91)
			CMV: higher susceptibility to CMV infection in solid-organ transplant recipients	(92)
			HIV: higher prevalence of AIDS-defining illness and lower CD4 lymphocytes levels	(93)
IFNLR1	rs10903035	A/G and G/G (A-major, G-Minor)	HIV/HCV: early treatment failure with HIV/HCV coinfected patients	(94)

*IFNL3, interferon lambda 3; IFNL4, interferon lambda 4; IFNLR1, interferon lambda receptor 1; HCV, hepatitis C virus; HTLV-1, human T-lymphotrophic virus type 1; HBV, hepatitis B virus; EBV, Epstein–Barr virus; CMV, cytomegalovirus; HSV, herpes simplex virus; ANDV, Andes virus; HIV, human immunodeficiency virus; PEG-IFN-α/RBV, pegylated-Interferon-α/Ribavirin*.

Modulation of IFNLR expression may have a great impact on the effects of a particular IFN-λ ligand, and thereby influence the subsequent signaling pathway and the outcome of infectious diseases. Multiple SNPs in the gene encoding IL28RA have been described (94–97). The rs10903035 SNP is located within the 3′UTR of the IL28RA mRNA sequence, suggesting a potential microRNA binding site. This particular SNP was identified as an independent risk factor for IFN-α treatment failure against hepatitis C virus (HCV) (44, 98). In addition, this SNP has been associated with insulin resistance in HIV/HCV coinfected patients (94). Another SNP in this gene, rs4649203, has been linked to the risk of psoriasis in four independent populations (96), and to the development of systemic lupus erythematosus (97). These observations suggest an important influence of IL28RA on infectious and autoimmune diseases.

Expression of IFN-λ ligands is modulated by SNPs in both transcription factor binding sites and methylation sites of the promoter region, as well as frameshift mutations (99–102). The IFN-λ gene layout is shown in Figure 2. The clinical impact of SNPs in the IFNL3/4 locus was originally observed in the context of IFN-α treatment outcomes in patients with chronic HCV (79, 80, 87, 90, 103). SNPs within this locus are in high linkage disequilibrium, e.g., rs12979860 with ss469415590 (103, 104), which complicates the exploration of the effects of individual SNPs. Therefore, the impact of some SNPs on IFN-λ expression is still debated. Most studies have concluded that the minor alleles of SNPs rs12979860 (CT/TT) and rs8099917 (TG/GG) are associated with reduced IFNL3 expression during chronic HCV infection, observed in liver biopsies (80, 105–107), serum, and PBMCs stimulated with polyI:C-, CMV-, and influenza virus (66, 76, 108, 109). However, it has also been shown that the TT allele of rs12979860 in hepatocytes expresses higher levels of IFNL1 and IFNL3 (110). This minor allele genotype of rs12979860 (TT) has also been associated with a higher and prolonged ISG expression in HCV infection (79, 80, 87, 90, 103, 111). Interestingly, the same SNP of the IFNL3 gene is associated with a higher ISG expression in mothers after childbirth, suggesting that postpartum the normalization of physiological control of IFN signaling depends on the IFNL3 genotype (112). Although the rs12979860 SNPs have been specifically associated with IFNL3/L4 expression, these SNPs might also affect the expression of the other IFN-λ genes (80, 87, 113).

The impact of the ss469415590 SNP on the expression of IFNL4 is, in contrast, very well described: in the context of a delta-G polymorphism, a frameshift mutation generates a gene containing an alternative reading frame, which causes IFNL4 to be functionally expressed in about 40% of Caucasians (90). An amino acid substitution at residue 70 of IFNL4 (P70S) decreases the antiviral activity *via* a reduction in the ISG expression levels (111).

Beside the impact of SNPs on innate immune signaling *via* differences in ISG expression profiles, an important impact on adaptive immune functions has been noted. We have shown that IFN-λ decreases virus-induced B-cell proliferation and antibody secretion in a dose-dependent manner. In addition, IFN-λ increases influenza-induced Th1 cytokines (IFN-γ, IL6), whereas influenza-induced Th2 cytokines decrease (IL4, IL5, IL9, IL13). These effects can also be reproduced with specific allelic combinations. In particular, the TG/GG allele of rs8099917 shows significantly lower levels of IFN-α, IL2, and IL6 secretion in influenza-stimulated PBMCs. In an influenza vaccine cohort, vaccine recipients with the rs8099917 TG/GG (minor) allele showed significantly higher vaccine-induced humoral immune responses (76). Similarly, in a cohort of children vaccinated against measles, the post-vaccine antibody titers were significantly higher in the group with the rs10853727 SNP AG and GG (minor allele) (89). Both SNPs rs8099917 and rs10853727 lie within the IFNL3 promoter region and have been associated with lower IFNL3 expression (76, 89).

## IFN-λ and Infectious Diseases

The dual role of IFN-λs, with direct antiviral effects (innate immunity) and more long-term immunomodulatory effects on T- and B-cell activation and modulation, can result in multiple possible interactions with different types of infectious disease. Table 2 summarizes the role of IFN-λs in several infectious diseases.

**Table 2:** **Described role of IFN-λσ in infectious diseases**.

Pathogens	Model	Role of IFN-λ	Reference
**Viruses**			
Cytomegalovirus (CMV)	*In vitro*: HFF cell line and stimulated peripheral blood mononuclear cells (PBMCs)	IFNL3 reduces CMV-induced CD4 T cell proliferation in PBMCs	(66)
	Clinical study		

Dengue virus	*In vitro*: DC and human lung epithelial cell line A549	IFNL1 induce CCR7 expression and DC migration upon dengue virus infection	(114)

HBV	*In vitro*: murine hepatocyte cell line (HBV-Met)	IFNL induces IFN-α/β-like antiviral response and inhibition of HBV replication in murine heptocyte cell line	(115)

Hepatitis C virus (HCV)	*In vitro: primary* hepatocytes and HUH7 cell lines.	IFNL induces type-1 interferon-like antiviral response and blocks HCV infection in human primary hepatocyte and HUH7 cells	(59, 115, 116)

HIV	*In vitro*: monocyte-derived macrophages	IFNL3 inhibits HIV infection of macrophage through the JAK-STAT pathway.	(117, 118)
	*In vitro*: T-cells and clinical study	IFNL induce antiviral state in culture primary T-cells and supress HIV-1 integration and posttranscriptional events	

HSV-1	*In vitro*: human lung epithelial cell line A549	Mediator complex (Med23) interacts with IRF-7 to enhance IFNL production and it inhibits HSV-1 replication	(119)
	Clinical study		

HSV-2	*In vitro*: human cervical epithelial cells	IFNL contributes to TLR3/RIG-1-mediated HSV-2 inhibition	(120)

Human metapheumovirus (HMPV)	*In vitro*: human lung epithelial cell line A549	Mice treated with IFNL prior to HMPV infection develop lower viral titer and reduced inflammatory responses	(121)

Influenza virus	*In vivo*: mice	IFNL restricts virus infection in epithelial cells of respiratory and gastrointestinal tracts	(122, 123)
	*In vitro*: cell lines	IFNL reduced Influenza A virus-induced disease, with less inflammatory side effects in comparison to IFN alpha	
	*In vivo*: infected mice		

Murine CMV	*In vitro*: intestinal epithelial cell lines	IFNL1 mediates antiproliferative and antiviral signals in intestinal epithelial cells	(59)

Norovirus	*In vivo*: infected mice	IFNL cures persistent murine norovirus infection	(124)

Lymphocytic chorimeningitis virus	*In vitro*: human lung epithelial cell line A549	IFNL2 showed more potent antiviral response to lymphocytic choriomeningitis virus than IFNL3	(125)

Rhinovirus	*In vitro*: human bronchial epithelial cell line (BEAS-2B)	Increased IFNL production reduces rhinovirus replication in bronchial epithelial cells	(126)

RSV	*In vitro*: primary human and mouse airway epithelial cells	TLR-s mediates IFNL production in primary airway epithelial cells and induces the antiviral response	(127, 128)
	*In vitro*: Hep-2 and Vero cells	IFNL-1 shows prophylactic potential against RSV	

Rotavirus	*In vivo*: infected mice	IFNL reduces viral replication in epithelia cells	(129)

SARS coronavirus	*In vitro*: human lung epithelial cell line A549	Ifnlr1^−/−^ mice exhibit increased susceptibility to SARS corona virus	(122, 130)
	*In vivo*: infected mice		

VSV	*In vitro*: mouse hepatocyte cell line	IFNL attenuates VSV replication in immortal mouse hepatocytes (MMHD3 cells)	(131)

West Nile virus	*In vitro*: Huh7.5 and HeLa cells	IFNL can efficiently prevent West Nile Virus infection in cell line	(67, 132)
	*In vivo: infected* mice	IFNL knockout animals show increased viral load in brain. Treatment with IFNL reduced blood–brain permeability for the virus	

**Bacteria**			
*Staphylococcus aureus* and *Pseudomonas aeruginosa*	*In vivo*: infected mice	Ifnlr1^−/−^ mice exhibits less pathology without changes in cell infiltrates	(133)

*Mycobacterium tuberculosis*	*In vitro*: human lung epithelial cell line A549	Induces IFNL expression on A549 lung epithelial cells	(134, 135)
	Clinical study	Observed increased concentration of IFNL2 in sputum of pulmonary tuberculosis patients	

*Listeria monocytogenes*	*In vivo*: infected mice	IFNL-mediated immune response may control bacterial colonization	(136)

*Salmonella typhimurium*	*In vitro*: human monocyte-derived macrophages	The activation of type III interferon by live and heat killed bacteria in phagocytic dentritic cells, but role in pathogenesis is not clear	(137)

*Borrelia burgdorferi*	*In vitro: stimulated* PBMCs	The ability of IFNL induction correlates with clinical isolates, type III IFN pathway in pathogenesis is yet to be determined	(138)

*HSV-1, herpes simplex virus-1; HSV-2, herpes simplex virus-2; RSV, respiratory syncytial virus; VSV, vesicular stomatitis virus; murine CMV, murine cytomegalovirus; SARS, severe acute respiratory syndrome*.

### Viral Infections

IFNs protect cells against viral infections. In response, every virus has evolved specific ways to counteract IFN signaling and its effects (139–143). Only a few studies have explored this in the context of IFN-λs. Parainfluenza virus 3 blocks antiviral mediators downstream of the IFNLR signaling by modulation of the STAT1 phosphorylation in BEAS 2B cells, a bronchial epithelial cell line (144). Dengue virus was recently shown to induce IFNL1 *via* its non-structural protein (NS1) in order to facilitate dendritic cell migration (114).

Using cell culture-based *in vitro* models, IFN-λs have been shown to play a role in controlling viral replication. In most studies, cultured cells were treated with IFN-λs and the impact of viral infection was assessed. These studies investigated human (66) and murine CMV (59), dengue virus (114, 145), encephalomyocarditis virus (28, 29, 146), herpes virus type 2 (120), hepatitis B virus (115), HCV (37, 60, 113, 115, 116, 147), HIV (40, 117, 118), human meta pneumovirus (121), influenza virus (122, 148–152), lymphocytic choriomeningitis virus (LCMV) (125), norovirus (124), respiratory syncytial virus (128, 153, 154), sendai virus (155–157), and vesicular stomatitis virus (131, 158, 159).

*In vivo*, the complexity of the role of IFN-λ within tissues and between various immune cells has been explored using an IL28RA^−/−^ mouse model, leading to the discovery of multiple important aspects of IFN-λ signaling (122, 130, 150).

A recent study by Lin et al. demonstrated that the effects of type III IFNs change with increasing age. Rotavirus was controlled by both type I and III IFN in suckling mice, whereas epithelial cells in particular were responsive. In adult mice, epithelial cells were responsive only to type III and not type I IFNs, suggesting an orchestrated spatial and temporal organization of the IFN-α and IFN-λ responses in the aging murine intestinal tract (160). However, there is some controversy regarding the rotavirus data, as other researchers have shown that rotavirus is specifically controlled by type III and not type I IFN (21518880). Mahlakoiv et al. showed that leukocyte-derived IFN-α/β and epithelial IFN-λ constitute a compartmentalized mucosal defense system to restrict enteric viral infection in mice. The authors concluded that epithelial barriers to IFN-λ may have evolved to reduce frequent triggering of IFN-α/β and thus reduce exacerbated inflammation (161). A study by Baldridge et al. showed that antibiotics could prevent the persistence of enteric murine norovirus infection, but only in the presence of functional IFN-λ signaling. The IL28RA^−/−^ mice showed a high rate of infection, despite the administration of antibiotics. This may suggest cross talk between the gut microbiota and IFN-λ signaling in modulating chronic viral infections (162). Important synergistic effects in the intestine have been described, with IL22-inducing IFN-λ expression in intestinal epithelial cells in a murine rotavirus infection model (163).

The role of IFN-λ during respiratory tract infections has also been explored using the IL28RA^−/−^ mouse model. The studies so far have concentrated on the classical role of IFNs as antiviral cytokines. The IL28RA^−/−^ mouse displayed a significantly higher burden of disease than wild-type mice during infections with influenza virus and SARS coronavirus (122, 130, 150). One study showed the immunoregulatory function of IFN-λ in an LCMV model. The authors noted that in an acute LCMV infection model, the IL28RA^−/−^ mouse showed a greater than normal CD4^+^ and CD8^+^ T-cell response compare to the wild type, whereas in a chronic LCMV infection model, the IL28RA^−/−^ mice showed a greater disease burden and a significantly reduced LCMV-specific T-cell response. The paper showed that germinal center B-cells were more frequent in peripheral blood in the IL28RA^−/−^ mice than wild-type mice. However, the LCMV-induced memory B-cell response, in terms of frequencies and LCMV-specific antibodies, was comparable (164).

The immunoregulatory actions of IFN-λs have been explored in an ovalbumin (OVA)-induced asthma model. The IL28RA^−/−^ mice showed a clear shift to increased Th2 cytokines and a more severe asthma phenotype. Importantly, IgE antibodies were also significantly increased (73). In this model, the IFNL2 (IL28A) immunoregulatory activity was dependent on lung CD11c^+^ dendritic cells to decrease OX40L, increase IL-12p70, and thereby promote Th1 differentiation (73). The potential role in infection-triggered asthma has also been explored in humans (72, 126).

Although these conclusions from mice studies are very important, a series of important differences to human effects have also been noted. In a human chimeric mouse model using human hepatocytes, the response rates of human and mice hepatocytes toward IFN-λs were very different, specifically in that mouse, hepatocytes did not respond to IFN-λ (56). In addition, the expression of IFNLR in immune cells seems to be strikingly different. Whereas B-cells in humans respond to IFN-λs, in B-cells from mice there seems to be no direct effect from IFN-λs (69, 164).

Studies on the impact of IFN-λs in clinical scenarios have been dominated by the strong association of IFNL3/L4 SNPs with spontaneous clearance of HCV and IFN-α treatment response (79, 80, 87, 90, 103, 111). Details on this important association have been reviewed in detail elsewhere (165–167). The association between IFN-λ SNPs and other infectious diseases is far less well explored. Not many studies have linked the genetic associations with mechanistic immunological assay.

Several studies have explored the association between SNPs in the IFNL3/L4 signaling and CMV replication. Transplant recipients with the rs8099917 GG allele demonstrate significantly less CMV primary replication. This SNP has been associated with reduced ISG expression upon infection (66). We postulate that this phenomenon has two reasons: (i) significant primary CMV replication is less likely due to a higher baseline ISG expression and (ii) naïve CMV-specific T cells from seronegative healthy blood donors show reduced proliferation capacity when pretreated with IFNL3 and stimulated with CMV lysate (66). In contrast, the rs368234815 ΔG SNP shows a higher risk for CMV retinitis in HIV-infected patients (91) and has been associated in a transplant cohort with an increased risk of CMV replication and disease, especially in patients receiving grafts from seropositive donors (92). Non-immunosuppressed patients with chronic periodontitis due to herpes virus infection show significant lower IFNL1 levels in gingival fluid compared to a healthy control group without viral replication (168), suggesting a protective effect of IFNL1 on virus replication, or CMV-induced antagonism of IFN-λ expression. These results highlight the different roles of IFN-λs in acute or chronic infection scenarios and viral reactivation.

The impact of IFN-λs on human T-cell leukemia type-1 virus has also been explored in several independent cohorts. The first evidence came from Kamihira et al. showing that the IFNL3 mRNA expression level was significantly higher in HTLV-1 mono-infection than HTLV-1/HCV coinfection. In addition, the high expression level was associated with the rs8099917 TT SNP (169). The impact of the rs8099917 GG SNP on the risk of HTLV-1 associated myelopathy/tropical spastic paraparesis (TSP) has since been confirmed (88). The impact of the rs12979860 SNP is more controversial. One study on the rs12979860 SNP showed that the CT/TT alleles were more frequent in patients with HTLV-1-associated myelopathy/TSP (81), although this finding was not replicated in two additional studies (170, 171). de Sa et al. reported that the major alleles of IFNL3 SNPs (rs12979860 CC and rs8099917 TT) are associated with a shift in the Th1/Th2 immune response toward a Th1 response (172). The Andes virus causes a hantavirus cardiopulmonary syndrome; in a cohort of Andes virus-infected patients, the minor alleles of rs12979860 and rs8099917 (TT and GG) were linked to milder disease compared to CT/CC and TG/TT (86).

The impact of the IFN-λ signaling on humoral immune function has been described in two vaccine cohorts: immunosuppressed patients vaccinated against influenza (76) and healthy children vaccinated against measles (89). These important observations hold promise for personalized vaccine strategies and adjuvant development (4).

### Bacterial Infections

The cytokine microenvironment of a tissue may have an impact on the rate at which a particular infectious bacterium can colonize and also influence the rate of infections. Planet et al. showed that IFN-λs might lead to important changes in the local microbiota during influenza infection. In a mouse model of influenza infection, the authors observed that mice with functional IL28 signaling showed more profound changes in their respiratory microbiota and subsequent higher colonization rates with *Staphylococcus aureus* compared to IL28RA^−/−^ mice (173). These important findings should be confirmed in a human cohort, as *S. aureus* is an important source of bacterial superinfection after an influenza infection. In addition, microbiota changes upon common clinical scenarios such as antibiotic treatment may be modulated by IFN-λs and their genotypes.

Bacteria including *M. tuberculosis* induce IFN-α/β and IFN-γ; however, little is known about the effects of IFN-λs in epithelial immunity. Gram-positive bacteria such as *S. aureus, Staphylococcus epidermidis, Enterococcus faecalis*, and *Listeria monocytogenes* induce IFN-λs, whereas *Salmonella enterica* serovar Typhimurium, *Shigella flexneri*, and *Chlamydia trachomatis* do not substantially induce IFN-λs, in intestinal and placental cell lines (134). Others have reported that *S. enterica* serovar Typhumurium can induce IFN-λs in human DCs (137). IFN-λ gene expression can be increased within DCs upon stimulation with bacterial components such as lipopolysaccharide. In particular, during *M. tuberculosis* infection, IFN-α plays an important regulatory role in the pathogenesis (12, 174). *M. tuberculosis* in A549 lung epithelial cells stimulates expression of IFN-λs. In addition, the IFNL2 concentration in sputum of patients with pulmonary tuberculosis is significantly higher than that in the sputum of healthy controls (135). Although the impact of IFN-λs has not been explored in more detail, the cross talk between IFN-α and IFN-λs may play a crucial role in the pathogenesis of *M. tuberculosis*. The modulation of Th1/Th2 toward Th1 may be of additional importance.

Neutrophil functions are crucial in clearing bacterial infections and wound repair (175, 176). A major target of the effects of IFN-λs may be neutrophils (62, 177). A study by Blazek et al. showed that in a collagen-induced arthritis model, IFNL1 showed anti-inflammatory function by reducing the numbers of IL17-producing Th17 cells and the recruitment of IL-1b expressing neutrophils, which is important to amplify the inflammatory process (62). Similar effects on neutrophil recruitment to the lung have been observed in an OVA-based asthma mouse model (73). Although somewhat speculative, this may suggest an important modulatory function of IFN-λs *via* neutrophil recruitment toward sites of bacterial infection.

So far, only one study has linked SNPs in genes involved in the IFN-λ signaling pathway with an increased risk of bacterial infections. Xiao et al. showed that SNP rs10903035 with G allele in the IL28RA was associated with significantly less frequent urinary tract infection (178).

### Parasite and Fungal Infections

The role of IFN-λs in parasitic and fungal disease has not yet been explored. Although somewhat speculative, helminth infections in particular might be regulated by SNPs in the IFN-λ system, considering the profound evidence on the importance of Th1/Th2 balance (6, 77, 78). Furthermore, for parasite infections of the liver such as *Plasmodium* spp. there is important evidence on the importance of the IFN-α signaling (13, 179–182). Due the regulatory interactions of IFN-α and IFN-λ and the clinical importance of relevant SNPs (31), it is not unreasonable to postulate an impact.

## Summary

IFN-λs, and their modulation *via* SNPs, are increasingly recognized as important players in a broad range of infectious diseases. Although the literature is still dominated by reports on HCV, work especially in mouse models has pointed out the important role in viral, respiratory, and gastrointestinal infections. Bacterial colonization and bacterial infections may also be modulated by IFN-λs. The important diversity in IFNs and the large number of SNPs adds a difficult-to-address layer of complexity. Therefore, further research on IFN-λs outside the HCV field is required to understand their roles and diagnostic and therapeutic potential. Most importantly, predictions of risks associated with infectious diseases have to be confirmed in independent cohorts to allow personalized medicine strategies.

## Author Contributions

Both the authors (AE and MS) have significantly contributed by writing the manuscript and designing the graphs.

## Conflict of Interest Statement

The authors declare that the research was conducted in the absence of any commercial or financial relationships that could be construed as a potential conflict of interest.
